# HcRed, a Genetically Encoded Fluorescent Binary Cross-Linking Agent for Cross-Linking of Mitochondrial ATP Synthase in *Saccharomyces cerevisiae*

**DOI:** 10.1371/journal.pone.0035095

**Published:** 2012-04-04

**Authors:** Lan Gong, Georg Ramm, Rodney J. Devenish, Mark Prescott

**Affiliations:** 1 Department of Biochemistry and Molecular Biology, Monash University, Clayton Campus, Victoria, Australia; 2 ARC Centre of Excellence in Structural and Functional Microbial Genomics, Monash University, Clayton Campus, Victoria, Australia; Université de Montréal, Canada

## Abstract

Genetically encoded fluorescent cross-linking agents represent powerful tools useful both for visualising and modulating protein interactions in living cells. The far-red fluorescent protein HcRed, which is fluorescent only in a dimer form, can be used to promote the homo-dimerisation of target proteins, and thereby yield useful information about biological processes. We have in yeast cells expressed HcRed fused to a subunit of mitochondrial ATP synthase (mtATPase). This resulted in cross-linking of the large multi-subunit mtATPase complex within the inner-membrane of the mitochondrion. Fluorescence microscopy revealed aberrant mitochondrial morphology, and mtATPase complexes isolated from mitochondria were recovered as fluorescent dimers under conditions where complexes from control mitochondria were recovered as monomers. When viewed by electron microscopy normal cristae were absent from mitochondria in cells in which mATPase complexes were cross-linked. mtATPase dimers are believed to be the building blocks that are assembled into supramolecular mtATPase ribbons that promote the formation of mitochondrial cristae. We propose that HcRed cross-links mATPase complexes in the mitochondrial membrane hindering the normal assembly/disassembly of the supramolecular forms of mtATPase.

## Introduction

In order to study and model cellular function it is necessary to exert control over specific processes or pathways, and monitor the outcomes. A range of approaches have been developed that allow events to be controlled or switched at the transcriptional, translational or post-translational level [1]. Modulating the spatial and temporal interactions of proteins post-translationally in live cells can be achieved in a number of ways including the use of cell permeable small molecules to control dimerisation domains coupled to proteins of interest or protein activation through intein splicing. These systems represent powerful tools for modulating cellular events. However, often they do not allow for the simultaneous monitoring of dimerization and biological effect. Hence there is considerable interest in developing new tools for such purposes. For example, it would be an advantage to be able to monitor the activity of a dimerising agent using a fluorescence readout.

Considerable effort has been expended in developing and optimising fluorescent protein technology, such that it is the most widely used technology platform for monitoring events in live cells [2]. Fluorescent proteins are readily engineered by genetic means and a considerable body of optical and structural data is available on a wide range of fluorescent protein variants. Here we investigate the possibility of using fluorescent proteins as a platform for developing cross-linking reagents.

The fluorescent protein HcRed was developed from a non-fluorescent tetrameric chromoprotein isolated from the sea anenome *Heteractis crispa* [3]. Amino acid substitutions were introduced around the chromophore to enhance fluorescence emission (λ^Em^_Max_, 645 nm; QY, 0.05) whilst other substitutions located at key positions on the surface of protein served to destabilise interactions between protomers converting the tetramer to a stable dimer. The post-translational chemistry required for synthesis of a fluorescent chromophore occurs only when the HcRed dimer is formed; monomers are not fluorescent [4]. The results of sedimentation velocity experiments indicated that HcRed was a dimer in solution over a range of concentrations with no evidence for the existence of monomers or tetramers [5]. The X-ray crystal structure of HcRed has been determined [5]. These properties suggest that HcRed would be useful as a fluorescent binary cross-linking agent for proteins of interest. Since only dimers are fluorescent, HcRed has the potential for monitoring the temporal and spatial formation of cross-linked products in live cells.

**Figure 1:**
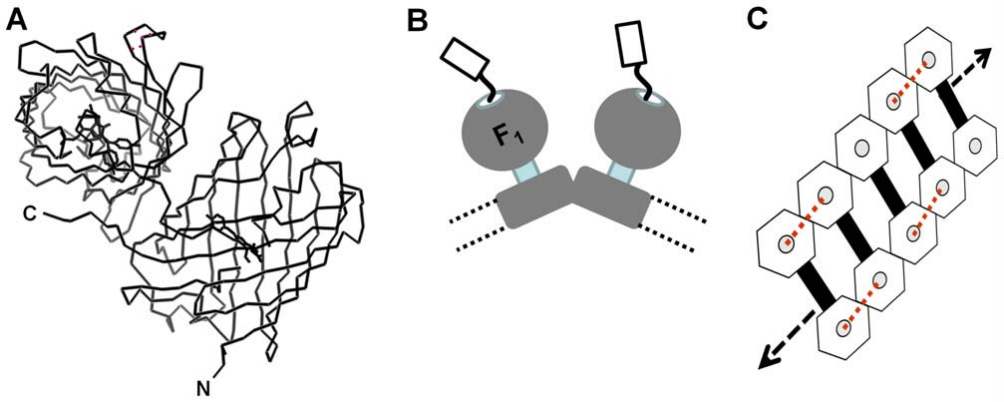
Using HcRed to crosslink ATP synthase complexes in the mitochondrial inner membrane. A. Fluorescent HcRed is an obligate homo-dimer. Representation shown in ribbon format was generated using PDB, 1YZW; [5]. The N- and C- termini are labelled for one of the two protomers. HcRed fused at its N-terminus to subunit γ of mtATPase promotes cross-linking of *neighbouring* mtATPase dimers in the membrane. B. Cartoon based on reported EM data showing for the inner membrane of the mitochondrion, the relative arrangement of each mtATPase monomer in dimer pairs. The C-terminus of subunit γ fused via a 25 amino acid polypeptide linker to an HcRed protomer is shown extending through the central pit of the F_1_ - sector and projecting into the matrix space. The distance between F_1_-sectors in these dimer pairs is too large for HcRed itself to form fluorescent dimers with a linker of this length. Broken lines represent the membrane boundaries. C. Dimer pairs of mtATPase embedded in the inner-membrane of the mitochondrion are organised into extended higher order assemblies resembling a ribbon. This organisation is important for proper cristae formation. Observed from the matrix and perpendicular to the inner membrane the catalytic sector which projects into the matrix space is represented by the hexagon shape from which the C-terminus of the γ-subunit protrudes from a central pit linked to an HcRed protomer (grey circle). Links at the level of the membrane between mtATPase complexes to form dimer pairs are shown by the black bar. The length of the linker between HcRed and subunitγ is sufficient to allow cross-links (rd dotted lines) to form between *neighbouring* dimers of mtATPase complexes, but not within dimer pairs.

In order to establish HcRed as a cross-linking agent we have used it to investigate the organisation of mitochondrial ATP synthase (mtATPase) in yeast cells. The assembly of mtATPase into linear supramolecular ‘ribbons’ in the inner membrane of the mitochondrion is believed to be a ubiquitous fundamental feature of intact active mitochondria [6]. mtATPase dimers represent the building blocks from which higher order linear assemblies or ‘ribbons’ are formed in the inner membrane of the mitochondrion which have been visualised using electron microscopy (for a recent review see [7]) (Fig. 1). The correct organisation of mtATPase in the inner membrane is believed to play a role in the formation and structure of the mitochondrial cristae [8]. The angled arrangement of monomer mtATPase into dimers promotes high membrane curvature. In yeast cells lacking expression of a subunit that facilitates mtATPase dimer formation this process is destabilised and dimers cannot be recovered *in vitro*; normal mitochondrial morphology and cristae are absent from such cells [9], [10]. Results of our published experiments showed *in vivo* cross-linking of mtATPase using DsRed fused to selected subunits of the complex resulted in a deranged mitochondrial morphology and elimination of cristae [11], [12]. However, DsRed is an obligate tetramer and a number of different cross-linked mtATPase oligomers were produced making it difficult to interpret the cross-linking data. Replacing DsRed with HcRed simplifies the outcomes of cross-linking. Our data using HcRed as a binary cross-linker indicates that it is the formation of cross-links between separate dimers of the supramolecular assembly disrupt both normal mitochondrial morphology and cristae formation suggesting that the ribbon structure undergoes continual reorganisation in the inner membrane of mitochondria.

## Materials and Methods

### Expression vectors, yeast strains and growth conditions

Details of yeast (*Saccharomyces cerevisiae*) strains used in this study are summarised in Table 1. YRD15 (*MATα, his3, ura3, leu2, [rho^+^]*) was the parental strain [13]. Strains lacking endogenous subunit λ and expressing γ-GFP or γ-DsRed fusions have been described elsewhere [12], [14]. Yeast strains lacking endogenous subunit γ and expressing γ-25-HcRed or γ-mRFP fusions (each with an intervening linker of 25 amino acids) were constructed by PCR mediated integration.

**Table 1:** Properties of yeast strains used in this study.

Yeast strain	Description	Cross-linking of mtATPase evidenced by CN-PAGE	Mitochondrial morphology observed by FM	Cristae formation observed by EM	Generation time (h)
YRD15	Parental strain (*MATα, his3, ura3, leu2, [rho^+^]*)	No	Wild-type reticulum	normal	6.98±0.25
mtGFP	Expresses GFP^1^ targeted to the mitochondrial matrix	No	Wild-type reticulum	ND	ND
γ-25-HcRed	Expresses γ-25-HcRed^2^	Dimers present in DM	Fragmented with appearance of balls and rings	reduced	10.08±0.33
γ-25-DsRed	Expresses γ-25-DsRed^2^	Dimers, trimers and tetramers cross-linking (data not shown; see also [11], [12])	Fragmented with appearance of balls and rings	reduced	13.09±0.88
γ-25-GFP	Expresses γ-25-GFP^2^ [14]	ND	Wild-type reticulum	ND	7.26±0.45
γ-25-mRFP	Expresses γ-25-mRFP^2^	No	Wild-type reticulum	normal	7.41±0.39
γ-25-HcRed-mtHcRed	Expresses the γ-25-HcRed^2^ and ‘free’ HcRed^1^ targeted to the mitochondrial matrix	Reduced levels of cross-linking in presence of free HcRed	Predominantly wild-type reticulum	ND	ND

1 expression from genomic location;

2 expression from vector. CN-PAGE, clear native polyacrylamide gel electrophoresis; FM, fluorescence microscopy; EM, electron microscopy; ND, not determined. Generation times are average of triplicate determinations +/− SD.

The series of yeast expression vectors used as DNA templates for the purpose of generating products for PCR integration were constructed as follows. A 217 bp DNA fragment encoding the *ADH1* terminator flanked by 5′ *Eag*I and 3′ *Bgl*II restriction sites was retrieved from *S. cerevisiae* genomic DNA and ligated into pGEM-T (Promega) to form pGEM-ADHT. A 1416 bp DNA fragment encoding the *Kluyveromyces lactis* URA3 selectable marker flanked by nested 5′ *Bam*HI and 3′ *Not*1 restriction sites was retrieved by PCR using *K. lactis* genomic DNA as template. The PCR product was ligated into the *Bgl*II/*Not*I sites of pGEM-ADHT to form pGEM-ADHT-KLURA3, respectively. An *Eag*1 fragment was recovered from pGEM-ADHT-KLURA3 and ligated into the single *Not*I site of pAS1NB-HcRedL, pAS1NB-mRFPL or pAS1NB-DsRED to form pAS1NB-HcRedL-ADHT-KLURA3, pAS1NB-mRFP-ADHT-KLURA3 and pAS1NB-DsRED ADHT-KLURA3. In this study DsRed was substituted by the fast maturing DsRed.T4 variant [15]. mRFP is a monomeric form of DsRed [16]. pAS1NB-HcRedL, pAS1NB-mRFPL or pAS1NB-DsRED.T4 were generated in an analogous fashion to pAS1NB-GFPL [17].

PCR products encoding HcRed, mRFP or DsRed.T4 [15], [16] linked to the yeast *ADH1* terminator and the *Kluyveromyces lactis URA3* selectable marker and bearing regions of homology sufficient for recombination with the yeast chromosomal *ATP3* gene (encoding mtATPase subunit γ) to generate fusions having a 25 amino acid linker (γ-25-DsRed.T4, γ-25-mRFP andγ-25-HcRed) were prepared using the relevant DNA template and the primers GACAAGCTGTCATTACTAATGAACTGGTTGATATTATTACTGGTGCTTCCTCTTTGGGAgatctaaacatgtctcgagc and ATGTTCTACAAAAACAACGTCAAATAAAGAGGCAATGCAGGGTGATTTTTTTATCAaccaccagtagagactagg where upper case indicates homology to site of homologous integration. Reactions were performed using Dynazyme EXT thermostable polymerase (Finnzymes). The products from 10 independent 50 µl reactions were pooled and incubated with 30 units of *Dpn*I for 2 h. PCR products were purified and concentrated using a single QIAquick PCR purification cartridge (Qiagen). Five µg of the DNA product were used to transform YRD15 cells by the method of Gietz *et al.* [18]. Transformants were selected by plating onto solid minimal medium supplemented with histidine and leucine. Individual URA^+^ transformant colonies were isolated and tested for growth on YEPE medium [19].

For targeting HcRed or GFP, not fused to another protein, to the mitochondrial matrix a DNA cassette encoding the first 55 amino acids of the yeast citrate synthase precursor polypeptide was retrieved by PCR from yeast genomic DNA as previously described [20] and ligated into the *Bgl*II cloning site of pAS1NB:YEGFP3L or pAS1NB:HcRedL to produce pAS1NB:citYEGFP and pAS1NB:citHcRed, respectively. To derive strains mtGFP and mtHcRed YRD15 cells were transformed with plasmids pAS1NB:citYEGFP and pAS1NB:citHcRed respectively. Growth media used were as described in Boyle *et al.* [21] and supplemented with uracil, histidine and leucine as required. Yeast cultures were grown aerobically in liquid medium (SaccE) containing 2% (v/v) ethanol at 28°C. All experiments used cells harvested in mid-logarithmic growth phase. The generation time (g) was calculated according to the following formula: g = (t/(3.3×log(B/b))), where t, b and B represent time interval in hours, cell number at the beginning of the time interval and cell number at the end of the time interval, respectively.

### Biochemical analyses

Mitochondria were prepared as described [9]. Whole cell lysates were prepared as described previously [22]. For immunoblotting mitochondria were boiled for 5 min, subjected to SDS-PAGE (12% polyacrylamide) and the proteins transferred to PVDF membranes (Pall Gelman Laboratory). Membranes were probed with rabbit subunit γ antisera (diluted 1∶1000) [11]. Blots were incubated with alkaline-phosphate conjugated secondary antibodies (Amrad-Pharmacia) and visualised using a Storm Phosphoimager (Molecular Dynamics) after incubation with chemifluorescent Vistra substrate (Amersham Pharmacia Biotech) [22].

Clear native polyacrylamide gel electrophoresis (CN-PAGE) was performed as described previously [12], [23]. Briefly, mitochondrial protein (10 µl of 20 µg/µl sample) was pelleted by centrifugation for 10 min at 100,000 g, and the pellet solubilised in 20 µl extraction buffer (50 mM NaCl, 2 mM 6-aminocaproic acid, 1 mM EDTA, 50 mM imidazole-HCl, pH 7.0, 5 mM phenylmethylsulfonyl fluoride, 4% (w/v) dodecyl β-maltoside (Roche Molecular Biochemicals). Where required dodecyl β-maltoside was replaced with digitonin (Sigma). The detergent/protein ratio was 4 g/g for both dodecyl β-maltoside and digitonin. Mitochondrial detergent extracts were centrifuged (100,000 g, 20 min) and supernatants loaded onto polyacrylamide gradient (3–13%) gels. Gels were imaged for fluorescence with a ProXPRESS multi-wavelength imager (PerkinElmer Life Sciences) using appropriate filters for excitation (DsRed/HcRed 540±25 nm) and emission (DsRed/HcRed 590±35 nm) [12].

### Fluorescence microscopy

Yeast strains were sampled during mid-logarithmic growth in SaccE medium for fluorescence microscopy. A 5 µl aliquot was sealed between a microscope slide and cover slip and immediately imaged using an Olympus FV500 confocal laser scanning microscope equipped with a 1.35 NA water immersion lens (Olympus 60×; UPlanapo). Image processing and analysis was performed using Olympus FluoView TIEMPO software (version 4.3) and the public domain software Image-J (version 1.36b) (http://rsb.info.nih.gov/ij/).

### Transmission electron microscopy

Transmission electron microscopy (TEM) was performed as described by Griffith *et al.* [24]. Briefly, yeast cells were cultured aerobically at 28°C to exponential growth phase in SaccE medium. Cells were fixed by the addition of an equal volume of double strength fixative (4% paraformaldehyde, 0.4% glutaraldehyde in PHEM buffer, 20 mM PIPES, 50 mM HEPES, pH 6.9, 20 mM EGTA and 4 mM MgCl_2_) to the growth medium and incubated for 20 min. Cells were harvested by gentle centrifugation, resuspended in single strength fixative and incubated for 2 h. Cells were washed in PHEM buffer and incubated for 1 h at room temperature in 1% (v/v) periodic acid in PHEM buffer. Cells were then washed three times in PHEM buffer before being embedded in 12% (w/v) gelatine for Tokuyasu cryo-sectioning. Ultrathin sections were retrieved by the direct pick-up method using a 1∶1 mixture of methylcellulose and 4% uranylacetate and transferred to formvar-coated EM grids (Electron Microscopy Sciences) [25]. The sections were air dried and visualised at 80 kV on a transmission electron microscope (Hitachi H-7500) equipped with a Gatan Multiscan 791 CCD camera.

## Results and Discussion

Subunit γ is a key component of the central rotor of mtATPase and essential for the growth of yeast cells on respiratory substrates including ethanol [14], [26]. Our published data show that mtATPase complexes containing subunit γ fused to the N-terminus of GFP via a linker of 25 amino acids (γ-25-GFP) are functional *in vivo* [14]. Molecular models indicated that the GFP moiety of such fusions protrudes through the central ‘pit’ located at the top of the F_1_-catalytic sector of mtATPase [14] (Fig. 1b). We here use a subunit γ fused to HcRed via a 25 amino linker (γ-25-HcRed) that functionally assembles into mtATPase complexes (Fig. 1b). Using our X-ray crystal structure for the fluorescent HcRed dimer [5] (Fig. 1a), structural data for yeast mtATPase [6], [27] and emerging data describing the organisation of mATPase [6], [28] in mitochondrial membranes, we have calculated that the γ-25-HcRed fusion protein has the potential to form cross-links over a maximum distance ranging between 114 Å and 174 Å. The precise distance would depend on the conformation of the polypeptide linker (ie. extended or α-helical). The distance between F_1_ heads within a population of mtATPase dimers in *S. cerevisiae* mitochondrial inner membranes as observed using EM was found to form a Gaussian distribution centred on 280 Å; no dimers were observed with a separation below 210 Å [6]. γ-25-HcRed is therefore incapable of forming cross-links within mtATPase dimers. However, γ-25-HcRed should be capable of forming cross-links between the F_1_-pits of *neighbouring* dimers we estimate to be separated by ∼150 Å (Fig. 1c; red dashed lines).

We first compared the growth rate of yeast cells expressing different γ-fusion proteins using ethanol as carbon source; conditions that require functional mtATPase complexes for growth (Table 1). The generation time for γ-25-HcRed cells (10.08±0.33 h) was increased compared to that observed for γ-25-GFP cells (7.26±0.45 h) and γ-25-mRFP cells (7.41±0.39 h) which each expressed γ fused to a fluorescent protein unable to dimerise, and control cells (YRD15; 6.98±0.25 h) in which γ was not fused to a fluorescent protein. Presumably the increased generation time for γ-25-HcRed cells results from binary cross-linking of *neighbouring* dimers induced by HcRed. The increased generation time for γ-25-DsRed cells (13.09±0.88 h) compared to γ-25-HcRed cells most likely reflects the ability of DsRed to generate sufficient ternary and quaternary cross-links [11], [12]. Collectively, these results suggest that HcRed fused to subunit γ modulates but does not prevent the synthesis of sufficient amounts of ATP for cell growth.

**Figure 2:**
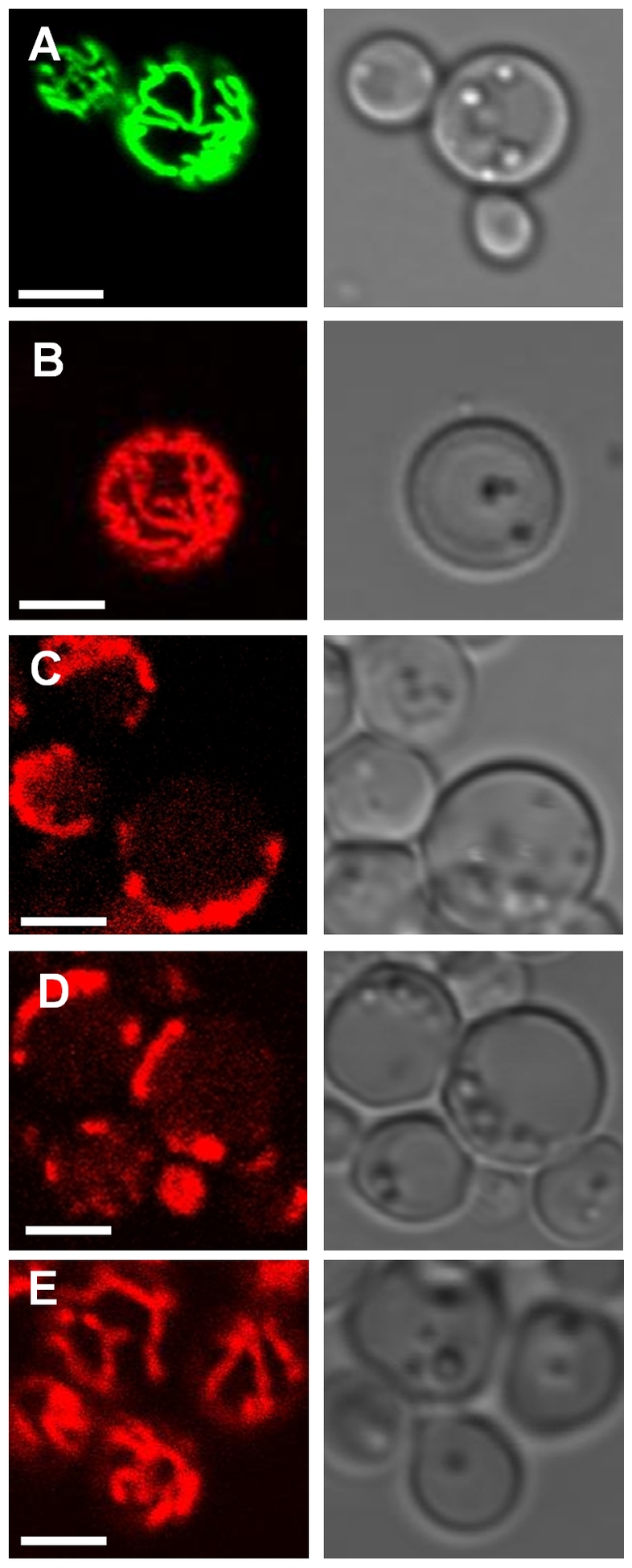
Yeast cells expressing γ-25-HcRed fusion proteins have abnormal mitochondrial morphology. Yeast cells were imaged for red or green emission using fluorescence microscopy. A, mtGFP; B, γ-25-mRFP; C,γ -25-HcRed; D, γ-25-DsRed, and E, γ-25-HcRed:mtHcRed. The corresponding DIC image is adjacent to the relevant fluorescence image. Scale bar = 2 µm.

Cells were next examined using fluorescence microscopy. Cells expressing GFP (mtGFP) targeted to the matrix but not fused to another protein and cells expressing mRFP fused to the C-terminus of subunit γ (γ-25-mRFP) both show a branched tubular network of mitochondria distributed around the periphery of the cell (Fig. 2 a & b respectively) characteristic of normal mitochondria during respiratory growth [11]. In contrast the filamentous mitochondrial reticulum was absent from γ-25-HcRed cells and replaced by puncta and ring-like structures, often aggregated to one side of the cell (Fig. 2c). The extended mitochondrial reticulum was also absent from γ-25-DsRed.T4 cells (Fig. 2d) expressing subunit γ fused to the tetrameric DsRed, an observation reported by us in a separate study [11], [12]. The mitochondrial reticulum was restored and appeared normal in γ-25-HcRed- mtHcRed cells (Fig. 2e) indicating that the availability of mtHcRed in the matrix was able to outcompete γ-25-HcRed *neighbouring* dimer formation, therefore suppressing cross-linking of mtATPase complexes by γ-25-HcRed. Individual mtATPase complexes in this strain will be associated with a HcRed dimer formed of a monomer contributed by mtHcRed and by HcRed tethered to the C-terminus of each γ subunit (γ-25-HcRed). This result confirms that the altered mitochondrial morphology characteristic of the γ-25-HcRed cells is a result of cross-linking of mtATPase complexes. Collectively these results suggest that cross-linking of mtATPase through subunit γ to form *neighbouring* dimers perturbs mitochondrial morphology.

**Figure 3:**
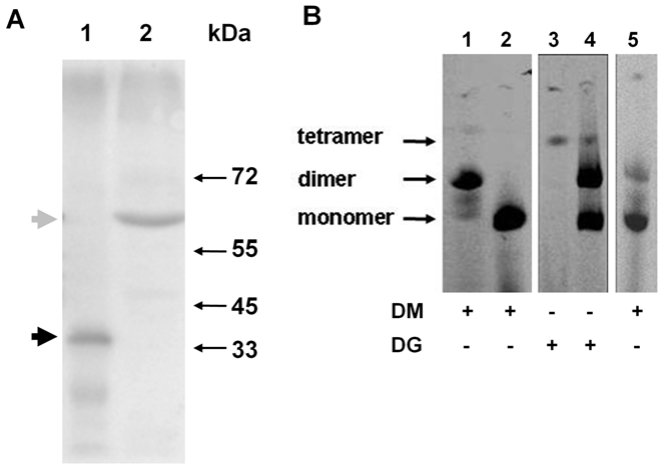
γ-25-HcRed fusion proteins crosslink mtATPase complexes. **A.** Mitochondrial lysates were subjected to SDS-PAGE and blots probed with antisera against mtATPase subunit γ. Lane 1, YRD15 control; and lane 2, γ-25- HcRed The position of molecular weight markers are indicated at right. The positions of the γ fusion protein and endogenous subunit γ are indicated at left by gray and black arrowheads, respectively. **B.** Mitochondria isolated from cells were solubilised with the addition of dodecyl β-maltoside (DM) or digitonin (DG) as indicated, and lysates subjected to clear native-PAGE. Gels were imaged for fluorescence: γ-25-HcRed (lanes 1 and 3); γ-25-mRFP (lanes 2 and 4), and γ-25-HcRed: mtHcRed (lane 5). The positions of monomer, dimer and tetramer mtATPase complexes are indicated at left.

We next investigated expression of γ fusion proteins by subjecting lysates of mitochondria isolated from yeast strains to SDS-PAGE and probing blots with antibodies against subunit γ (Fig. 3a). A major band was observed in a lysate of γ-25-HcRed mitochondria (Fig. 3a, lane 2) with a mobility (M_r_∼58,000) corresponding to the expected size of the fusion polypeptide (56,700 Da). The position of subunit γ not fused to another protein was observed in lysates of control YRD15 mitochondria (Fig. 3a, lane 1) with a mobility (M_r_∼32,000) corresponding to the expected size (30,661 Da). These results indicated that γ-25-HcRed fusion protein was expressed at the expected size with minimal degradation.

Associations between mtATPase complexes within mitochondria can be readily preserved and analysed using CN-PAGE techniques [11], [23], [29]. We next used detergent solubilisation to distinguish between dimer and monomer formation. Dimer and monomer forms of mtATPase can be retrieved using the detergents dodecyl β-maltoside (DM) or digitonin (DG), respectively to solubilise isolated mitochondria [9]. mtATPase complexes recovered from lysates of mitochondria isolated from cells expressing γ fusion proteins (Table 1) were subjected to CN-PAGE (Fig. 3b) and imaged for red fluorescence emission. When solubilised with DM mtATPase complexes were recovered from γ-25-HcRed mitochondria predominantly as dimers (Fig. 3b, lane 1). In contrast, mtATPase solubilised from γ-25-mRFP mitochondria using DM was recovered only as monomers (Fig. 3b, lane 2). These results indicate that HcRed promotes the isolation of dimers under conditions where mtATPase complexes would otherwise be recovered as monomers. Furthermore, the complexes solubilised from γ-25-HcRed mitochondria with DM were shown to retain ATPase activity by using an *in situ* ATPase assay (data not shown). We next investigated whether the dimerisation of mtATPase promoted by γ-25-HcRed fusions could be prevented by the expression, in the mitochondrial matrix, of HcRed not fused to another protein as suggested by fluorescence microscopy (Fig. 2e). Indeed, predominantly monomers were recovered from γ-25-HcRed:mtHcRed mitochondria solubilised with DM (Fig. 3b, lane 5) indicating that the presence of mtHcRed not fused to another protein can suppress cross-linking.

When γ-25-HcRed mitochondria were solubilised with DG (Fig. 3B, lane 3) only a small amount of fluorescent material corresponding to a tetramer was recovered. This result suggests that γ-25-HcRed cross-links *neighbouring* dimers (not within dimer pairs) under conditions that otherwise retrieve dimer pairs. Higher order assemblies of mtATPase (tetramers and larger) are not as efficiently solubilised from mitochondria using DG. As expected dimers, presumably representing dimer pairs, were recovered from γ-25-mRFP mitochondria solubilised with DG (Fig. 3b, lane 4).

**Figure 4:**
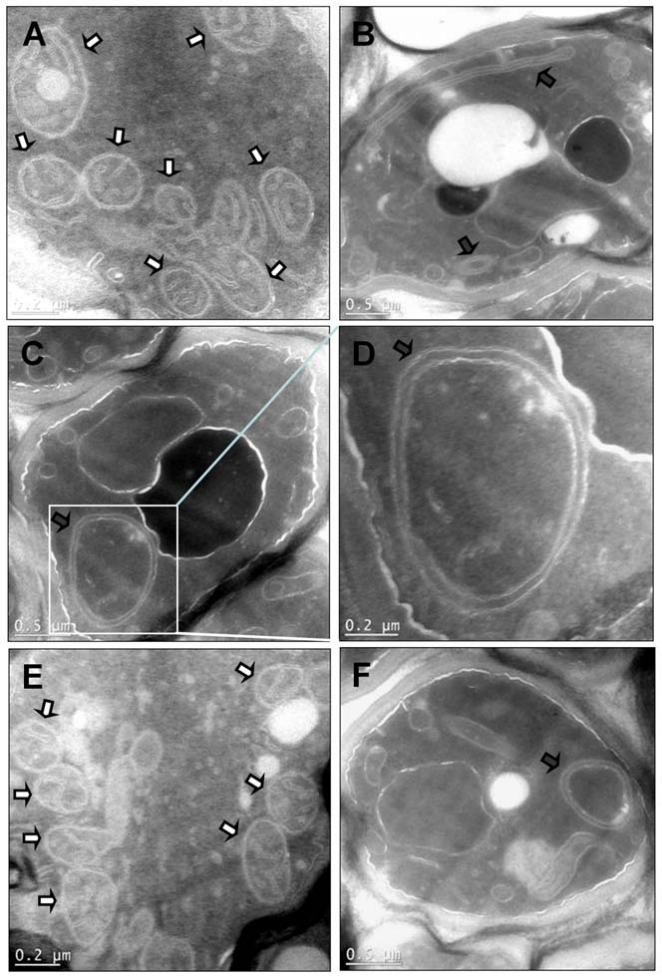
Mitochondria in yeast cells expressing γ-25-HcRed fusion proteins lack cristae. Transmission electron microscopy was performed on cell sections. A, YRD15; B - D, γ-25-HcRed; E, γ-25-mRFP, and F, γ-25-DsRed. Boxed area in C is shown magnified in D. Mitochondria and abnormal mitochondria are highlighted by white and black arrows respectively. Scale bars are shown.

Finally, we examined yeast mitochondrial ultrastructure using transmission electron microscopy (Fig. 4). The mitochondria in YRD15 control cells showed well-developed morphology characterised by the presence of numerous cristal membranes (Fig. 4a). The mitochondria in γ-25-HcRed cells showed a defect in cristal formation and were often very elongated lacking an obvious matrix space (Fig. 4b), or large and swollen (Fig. 4c & d). Similar observations were made for mitochondria in γ-25-DsRed cells (Fig. 4f) consistent with our previous observations [11]. Cells expressing γ-25-mRFP showed mitochondria with normal morphology and having numerous well-developed cristae (Fig. 4e). These results suggest that the binary cross-linking of mtATPase complexes promoted by expression of γ-25-HcRed fusion leads to elimination of mitochondrial cristae.

In a previous study we used data obtained using a γ-25-DsRed fusion protein as a cross-linking agent to conclude that correct arrangement of mtATPase complexes is essential for normal cristae formation [11]. However, DsRed produced a range of concatenated cross-linked products limiting our interpretation of the data. In the present study we show, using the self-limiting nature of the binary cross-linker HcRed, that cross-linking between *neighbouring* dimers in the ribbon assembly leads to loss of normal mitochondrial morphology and cristae (Fig. 1, Table 1). It is generally agreed that oligomer formation occurs by packing of dimer pairs to form helical mtATPase oligomers that wrap the tubular cristae as originally proposed by Allen *et al.* [8]. Mitochondria are highly dynamic organelles that undergo continuous fission and fusion. It is presumed such events require a highly coordinated disassembly and reassembly of the long helical assemblies of mtATPase. Such reorganisation would be compromised if crucial interfaces between mtATPase complexes are cross-linked as is the case in γ-25-HcRed cells.

The use of this probe is not limited to cross-linking of mtATPase complexes and we anticipate others will find it useful in their own studies. It is useful to be aware of potential limitations of using HcRed. Compared to many other fluorescent proteins the post-translational chemistry to form a fluorescent HcRed is relatively slow. Although not reported in the literature it may have a t_0.5_ for maturation of several hours. Specific interactions (ie. cross-linking) between protomers will form within this time period once the characteristic β-barrel has folded. This property will determine the earliest point at which cross-linking might be expected to occur if conditional expression systems are to be used.

Fluorescent proteins are a platform for a wide-range of reporters capable of monitoring cellular events. We envisage further developments in the technology that would encompass fluorescent proteins able to undergo reversible cross-linking driven by illumination with specific wavelengths of light. The mechanism of photo-switching in fluorescent proteins involves a reversible *cis-trans* isomerisation of the chromophore together with a coordinated movement of surrounding amino acid side-chains (see for review [30]). It is possible that such rearrangements might be harnessed to modulate dimer formation. Optogenetic tools are contributing to important advances in our understanding of cell biology (see for review [31]); an approach to modulate interactions using focussed light would make a worthwhile addition to the tool box.
